# Emerging variants of concern in SARS-CoV-2 membrane protein: a highly conserved target with potential pathological and therapeutic implications

**DOI:** 10.1080/22221751.2021.1922097

**Published:** 2021-05-09

**Authors:** Lishuang Shen, Jennifer Dien Bard, Timothy J. Triche, Alexander R. Judkins, Jaclyn A. Biegel, Xiaowu Gai

**Affiliations:** Children’s Hospital Los Angles, Department of Pathology and Laboratory Medicine, Keck School of Medicine of University of Southern California, Los Angeles, CA, USA

**Keywords:** SARS-CoV-2, membrane protein, M:I82T mutation, genomic surveillance, variant of concern, COVID-19

## Abstract

Mutations in the SARS-CoV-2 Membrane (M) gene are relatively uncommon. The M gene encodes the most abundant viral structural protein, and is implicated in multiple viral functions, including initial attachment to the host cell via heparin sulphate proteoglycan, viral protein assembly in conjunction with the N and E genes, and enhanced glucose transport. We have identified a recent spike in the frequency of reported SARS-CoV-2 genomes carrying M gene mutations. This is associated with emergence of a new sub-B.1 clade, B.1.I82T, defined by the previously unreported M:I82T mutation within TM3, the third of three membrane spanning helices implicated in glucose transport. The frequency of this mutation increased in the USA from 0.014% in October 2020 to 1.62% in February 2021, a 116-fold change. While constituting 0.7% of the isolates overall, M:I82T sub-B.1 lineage accounted for 14.4% of B.1 lineage isolates in February 2021, similar to the rapid initial increase previously seen with the B.1.1.7 and B.1.429 lineages, which quickly became the dominant lineages in Europe and California over a period of several months. A similar increase in incidence was also noted in another related mutation, V70L, also within the TM2 transmembrane helix. These M mutations are associated with younger patient age (4.6 to 6.3 years). The rapid emergence of this B.1.I82T clade, recently named Pangolin B.1.575 lineage, suggests that this M gene mutation is more biologically fit, perhaps related to glucose uptake during viral replication, and should be included in ongoing genomic surveillance efforts and warrants further evaluation for potentially increased pathogenic and therapeutic implications.

## Introduction

Genomic surveillance is critical for identification of SARS-CoV-2 variants of concern (VOCs) [1]. Children’s Hospital Los Angeles (CHLA) has routinely sequenced all viral isolates from over 2900 pediatric and adult COVID-19 cases since March 2020. Using the CHLA COVID-19 Analysis Research Database (CARD), we have routinely performed local genomic epidemiology and genomic surveillance of viral sequences submitted to GISAID and NCBI GenBank [2–4]. This allowed us to identify SARS-CoV-2 haplotypes and their localized transmission patterns that arose early and became dominant [5], specifically the D614G S spike protein mutation that was unidentified prior to April but which was identified in 99.3% of viral isolates from our pediatric COVID-19 patients by June of 2020 [6]. We also identified the potential association of phylogenetic clade 20C with more severe pediatric disease [6]. Here we report a new VOC with a signature mutation in the M protein gene, an otherwise overlooked but potentially significant site of increasing numbers of mutations, reminiscent of accumulating mutations in the Spike gene of previously reported VOCs, such as B.1.1.7 and B.1.351.

## Materials and methods

### Ethics approval

Study design conducted at Children’s Hospital Los Angeles was approved by the Institutional Review Board under IRB CHLA-16-00429.

### SARS-CoV-2 whole genome sequencing

Whole genome sequencing of the 2900 samples previously confirmed at Children’s Hospital Los Angeles to be positive for SARS-CoV-2 by reverse transcription-polymerase chain reaction (RT-PCR) was performed as previously described [6].

### SARS-CoV-2 sequence and variant analysis

Full-length SARS-CoV-2 sequences have been periodically downloaded from GISAID and NCBI GenBank. They are combined with sequences from CHLA patients, annotated, and curated using bioinformatics tools previously described [5].

### Phylogenetic analysis

Phylogenetic analysis was conducted using the NextStrain phylogenetic pipeline (version 3.0.1) (https://nextstrain.org/). Mafft (v7.4) was used in multiple sequence alignment [7], IQ-Tree (multicore version 2.1.1 COVID-edition) and TreeTime version 0.7.6 were used to infer and time-resolve evolutionary trees, and reconstruct ancestral sequences and mutations [8, 9].

### Protein structure prediction

M protein structural predictions were carried out using the Missense3D service hosted online by the Imperial College London (http://www.sbg.bio.ic.ac.uk/~missense3d/) [10].

## Results

**Figure 1.**
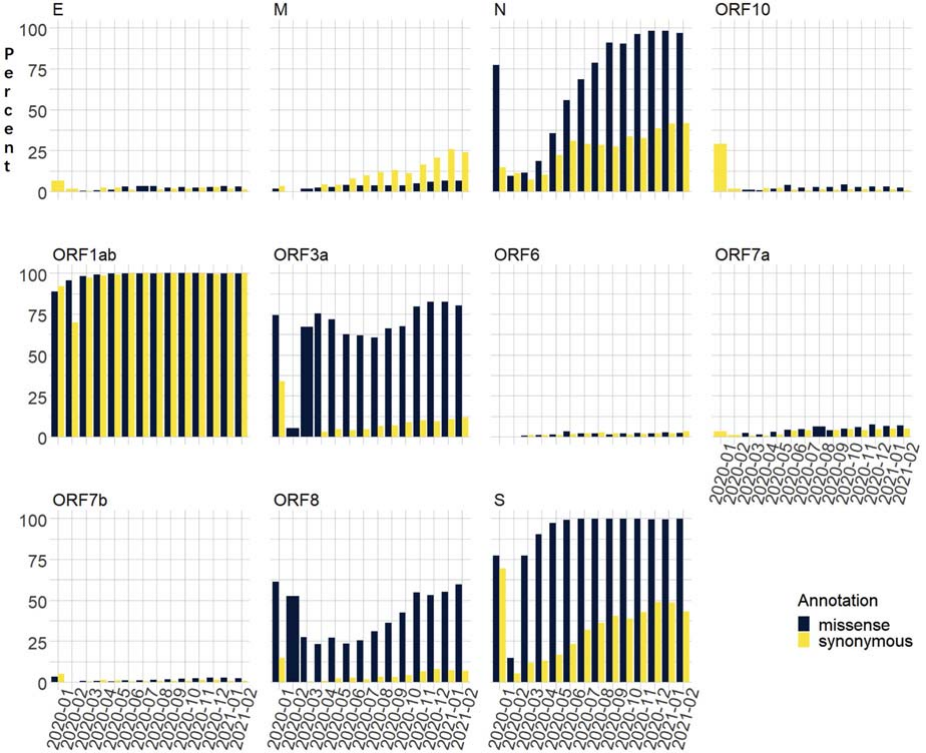
Percent of missense or synonymous mutations in viral isolates from USA over 14 months.

We evaluated 143,609 USA SARS-CoV-2 viral genomes, including 2900 from our own patients, and 622,033 global viral genomes, reported to GISAID and NCBI GenBank through late-February 2021. By measuring the ratios of the genomes carrying at least one missense mutation and genomes carrying at least one synonymous mutation in comparison to the reference genome (NC_045512) we identified distinctively different mutation profiles over time across SARS-CoV-2 genes (Figure 1). To evaluate these profiles, we performed exhaustive permutations of all possible changes at each base pair position of the SARS-CoV-2 gene to estimate missense mutations that could occur by chance. Using this approach, we estimated missense mutations should occur at least 2.7 times more frequently than synonymous mutations in the Envelope (E) gene, 3.1 times more frequently in the M gene, and as much as 3.8 times in ORF6 gene.

However, only the M gene has a ratio of missense to synonymous mutation carrying genomes consistently below 1.0 since the beginning of the pandemic. Further, this ratio has generally decreased over time. Among viral genomes from the USA as of late-February 2021, 6616 isolates (4.6%) carried missense and 22,908 (16.0%) carried synonymous mutations in the M gene, for a ratio of 0.29. While 116,005 genomes showed no M mutations of any kind, a small number of the genomes, 1920 to be precise, carried both missense and synonymous mutations. Globally, the ratio is even lower for the same period: there were 29,431 (4.73%) missense, and 197,205 (31.7%) synonymous mutations in the M gene, for a ratio of 0.149. This suggests that the M gene is highly conserved and potentially under strong purifying selection. It is thus of great potential interest that the incidence of some missense M gene mutations has recently increased over 100 fold in the past four months and continues to increase. The reason for this remains unclear but may suggest an underlying biologic advantage yet to be identified.

Other SARS-CoV-2 genes, notably the S (spike) gene and the large ORF1ab gene, appear to be tolerant of missense mutations, with multiple mutations in virtually every isolate. Indeed, some Spike missense mutations, like D614G and E484K, are advantageous, leading to rapid spread and increased frequency in the overall population and the emergence of a number of VOCs that uniformly include mutations like the D614G, which is now found in nearly every isolate worldwide but was unreported a year ago [11, 12]. A different pattern is evident in ORF1ab where the relatively large size of this gene coupled with mutational tolerance has led to essentially all isolate showing one or more mutations in ORF1ab, producing a ratio close to 1.

For each M gene missense mutation, we calculated the percentage of mutation-carrying viral genomes at country and in USA state levels, and then compared the frequency increase vs. previous months, to determine the timing and fold increase. The M gene is relatively silent, with only 4 missense mutations that each account for 0.4% or more of the global viral genomes in the month of February 2021 (M:A2S at 1.01%, M:V70L at 1.004%, M:I82T at 0.68%, and M:F28L at 0.41%). However, the percentage of viral genomes carrying missense M mutations has increased over time, and the accumulation of some mutations in the M protein appear to have surged recently both in the USA and globally. In the USA, 2.21%, 3.66% and 5.96% of reported viral genomes in April, August and December 2020 had missense M mutations. There has been a sharp increase in these missense mutations over the last three months, rising to 6.6% in the USA by February 2021 (Figure 1).

**Figure 2.**
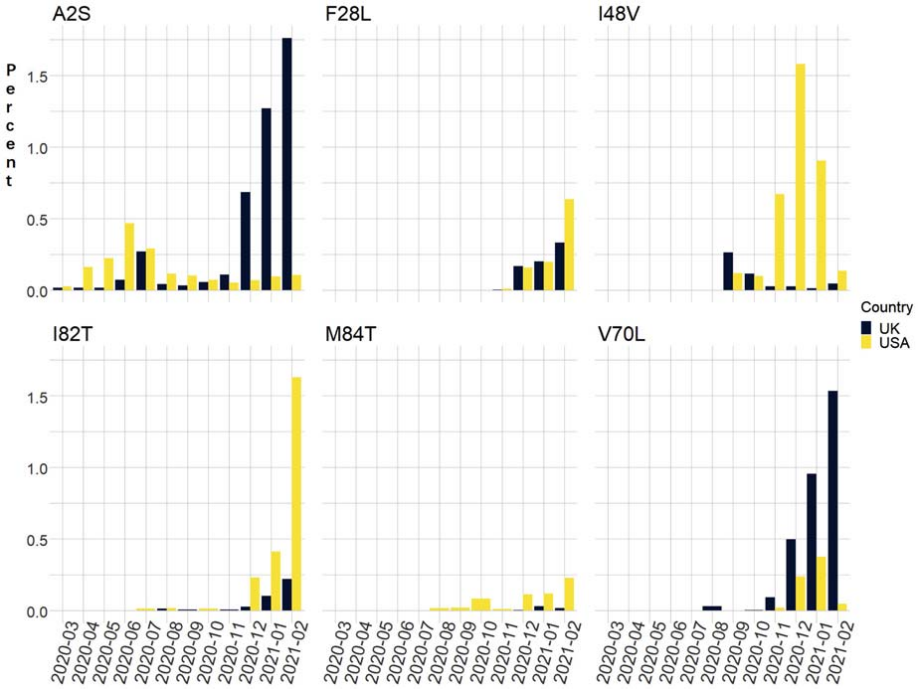
Percent of specific M mutations in viral isolates from USA and UK over 14 months.

We identified six mutations which showed a significant increase in frequency, reaching 0.4% during the recent months and which could potentially account for this acceleration of M mutations (Figure 2). The M:I48V mutation is highly specific to the USA at 1.18% in January 2021 (Table 1, Table S1), 87 times that observed in samples from outside the USA (0.014%). Most (78.3%) of the mutation-carrying isolates belong to the B.1.375 lineage (Table S3). This mutation also shows considerable geographic variability, with the greatest frequency in isolates from the northeast and along the East coast: Rhode Island – 68.8%; Connecticut – 24.0%; New Hampshire – 17.8%; Florida – 15.9%; Massachusetts – 15.7%; Tennessee – 11.8%; Arkansas – 11.1% as of December 2020. Over the last three months, 129 of the 293 viral sequences from Rhode Island had the same M:I48V missense mutation, an approximately 9.5 fold higher frequency than in other USA locations. After peaking in December, the M:I48V missense mutation appear to be diminishing with a current 0.13% frequency in the USA (Table 1, Table S1, Figure 2).

**Table 1.** Summary of the frequencies of M gene missense mutations in isolates over 14 months.

Mutation	Month	# in USA	% in USA	% Non-USA	% World
M:V70L	2021-02	5	0.047	1.255	1.004
M:V70L	2021-01	137	0.373	0.745	0.642
M:V70L	2020-12	41	0.237	0.302	0.288
M:V70L	2020-11	2	0.02	0.064	0.057
M:F28L	2021-02	67	0.631	0.355	0.413
M:F28L	2021-01	72	0.196	0.186	0.189
M:F28L	2020-12	27	0.156	0.115	0.124
M:F28L	2020-11	1	0.01	0.004	0.005
M:I82T	2021-02	172	1.62	0.435	0.683
M:I82T	2021-01	150	0.408	0.223	0.275
M:I82T	2020-12	40	0.231	0.043	0.082
M:I82T	2020-10	1	0.014		0.002
M:I82T	2020-08	1	0.016	0.004	0.007
M:I82T	2020-07	1	0.012	0.006	0.008
M:I48V	021-02	14	0.132	0.037	0.057
M:I48V	2021-01	331	0.901	0.008	0.255
M:I48V	2020-12	275	1.592	0.02	0.345
M:I48V	2020-11	67	0.669	0.016	0.118
M:I48V	2020-10	7	0.097	0.075	0.078
M:I48V	2020-09	6	0.119	0.153	0.148
M:I48V	2020-01	1	1.613		0.148
M:M84T	2021-02	24	0.226	0.032	0.073
M:M84T	2021-01	43	0.117	0.025	0.05
M:M84T	2020-12	19	0.11	0.017	0.036
M:M84T	2020-11	1	0.01	0.009	0.009
M:M84T	2020-10	6	0.083		0.011
M:M84T	2020-09	1	0.02	0.004	0.006
M:M84T	2020-08	1	0.016	0.086	0.072
A2S	2021-02	11	0.104	1.248	1.01
A2S	2021-01	35	0.095	0.871	0.657
A2S	2020-12	12	0.069	0.467	0.384
A2S	2020-11	5	0.05	0.093	0.087
A2S	2020-10	5	0.07	0.055	0.057
A2S	2020-09	5	0.1	0.049	0.057
A2S	2020-08	7	0.115	0.289	0.253
A2S	2020-07	24	0.289	1.174	0.888
A2S	2020-06	52	0.467	0.349	0.401
A2S	2020-05	18	0.223	0.007	0.082
A2S	2020-04	19	0.158	0.039	0.069
A2S	2020-03	3	0.023	0.008	0.012

**Figure 3.**
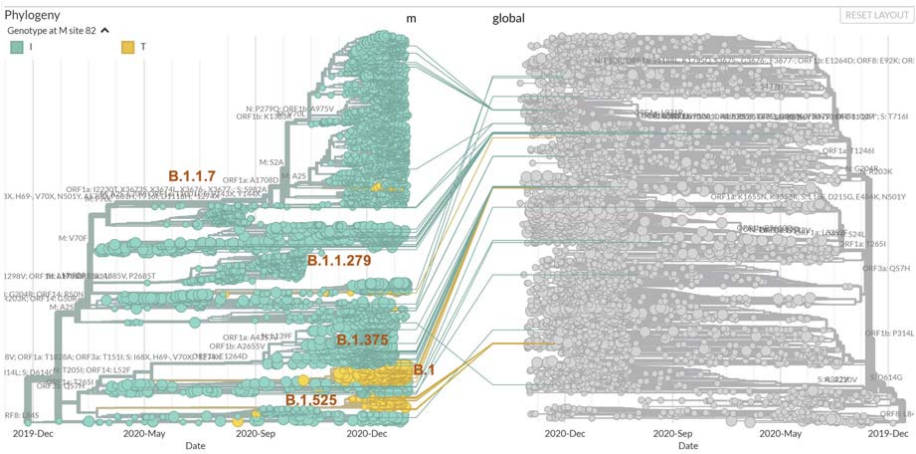
Phylogenetic tree of viral genomes carrying missense M mutations (left), coloured by the genotypes at M:82 (I: green; T: yellow), overlaid on the global SARS-CoV-2 phylogenetic tree (right and grey).

**Figure 4.**
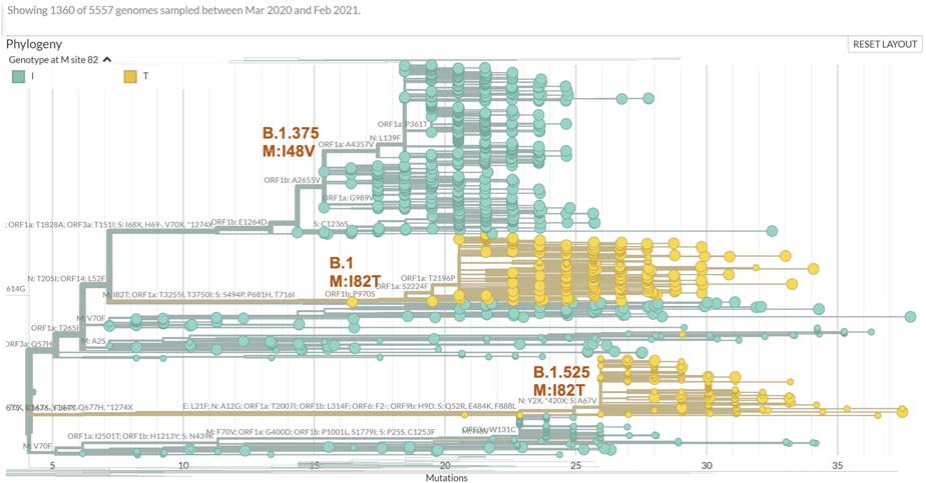
Detailed view of phylogenetic tree of viral genomes carrying missense M mutations coloured by the genotypes at M:82 (I: green; T: yellow), demonstrating proposed new clade B.1 M:I82T (middle), falling between B.1.375 clade that carries M:I48V (top) and B.1.525 clade that also carries M:I82T (bottom).

In contrast, the M:I82T mutation increased in frequency 116 fold from 0.014% in October 2020 to 1.62% in February 2021 in the USA and continues to grow. While it predominately circulated in New York and New Jersey, over the past 2 months M:I82T has surged outside the USA including Aruba (5.2%) and Nigeria (33.1%) (Table 1, Table S1). This mutation presents mainly within the B.1 (44.0%) and B.1.525 (38.1%) lineages. Currently, 99.7% of the B.1.525 lineage isolates carry the M:I82T mutation. While this mutation is scattered across multiple phylogenetic clades, most cases cluster in two recent clades (Figures 3 and 4), suggesting a likely selective advantage in certain haplotype backgrounds.

The largest M:I82T carrying clade is part of a young M:I82T sub-B.1 lineage that has surged over the past 3 months to account for 14.4% of B.1 lineage isolates in February, and now constitutes 0.7% of all B.1 lineages. There were 10 other missense mutations present in at least 90% of the isolates in this clade and 8 of them were enriched by 73 to 146 fold compared to the general B.1 lineage including the 3 signature mutations in the spike protein (S:S494P, the S:P681H and S:T716I) found in the B.1.1.7 lineage (Table 2). Thus, the M:I82T clade is significantly phylogenetically separated from other B.1 lineage clades, and may deserve consideration for a separate lineage designation (Figures 3 and 4).

**Table 2.** Potential signature mutations in the B.1 sub-clade that carries the M:I82T mutation.

# Isolates in clade	Mutations	Gene	Aino acid change	Annotation	# Isolates w/mutation	% Isolates in clade w/mutation
348	T26767C	M	I82T	missense	348	100
348	C28887T	N	T205I	missense	341	97.99
348	T23042C	S	S494P	missense	332	95.4
348	C23709T	S	T716I	missense	316	90.8
348	C23604A	S	P681H	missense	336	96.55
348	A23403G	S	D614G	missense	347	99.71
348	G25563T	ORF3a	Q57H	missense	332	95.4
348	A6851C	orf1ab	T2196P	missense	174	50
348	C16375T	orf1ab	P5371S	missense	328	94.25
348	C6936T	orf1ab	S2224F	missense	215	61.78
348	C11514T	orf1ab	T3750I	missense	327	93.97
348	C10029T	orf1ab	T3255I	missense	328	94.25
348	C1059T	orf1ab	T265I	missense	336	96.55
348	C14408T	orf1ab	P4715L	missense	343	98.56
348	A1180G	orf1ab	P305P	synonymous	317	91.09
348	T20748C	orf1ab	Y6828Y	synonymous	329	94.54
348	A1180G	orf1ab	P305P	synonymous	317	91.09
348	T20748C	orf1ab	Y6828Y	synonymous	329	94.54
348	C6730T	orf1ab	N2155N	synonymous	260	74.71
348	C3037T	orf1ab	F924F	synonymous	345	99.14
348	C241T	5UTR_orf1ab		upstream_gene	343	98.56
348	C29719T	ORF10_3UTR		intergenic	318	91.38

This new sub-B.1 clade warrants close surveillance given the similarity to the initial patterns of B.1.1.7 and B.1.429, which quickly became the dominating lineages in Europe and California, respectively. The second largest M:I82T carrying clade arose only recently in December 2020 and is mainly circulating in Europe and Africa, where it was co-segregating with the S:E484K Spike protein mutation, forming lineage B.1.525 (Table S2, https://cov-lineages.org/global_report_B.1.525.html). This clade is smaller than the M:I82T clade in the USA, as described above, which lacks the S:E484K mutation. This suggests that M:I82T may confer a biologically selective advantage independent of the S:E484K, a known predictor of more severe viral infection. Another mutation carried by most isolates in this clade (98%) that is worth noting is N:T205I, because it is present in multiple VOCs including CAL.20C (B.1.429 and B.1.427) and B.1.351, and that M and N proteins are both important for viral assembly. The novel combination of M:I82T, the three signature Spike mutations (S:S494P, the S:P681H and S:T716I) from B.1.1.7, and the N:T205I mutation is therefore of particular concern.

Other M gene mutations have also increased in frequency. In the UK, M:V70L first appeared in September 2020 and the frequency increased 382 fold from 0.004% in October to 1.5% in February 2021, when it was also present in Switzerland at 3.6% and in Belgium at 3.0%. The M:V70L-carrying virus isolates are part of the minor lineages under the B.1.1.7 lineage. Another mutation in the same codon, M:V70F, has persisted at low frequencies across multiple countries since March 2020. M:F28L first appeared in November 2020 and is highest in Austria (02/2021, 39.3%), Ghana (01/2021, 6.4%) and Japan (01/2021, 2.3%) but also observed in Spain (2.1%), Belgium (0.4%), and the Netherlands (0.8%), by February 2021. Within the USA (0.6%), this mutation is present at high levels in isolates from Virginia (13.1%) and Maryland (3.8%). It presents mainly within the R.1 (34.2%) and B.1.1.7 (48.9%) lineages, with 98% of the R.1 lineage isolates carry the M:F28L as a signature mutation. M:A2S has existed widely across the world since last March, peaked to 0.9% in July globally, and has re-emerged globally at 1.0%, with levels of up to 3.22% in Spain and 1.76% in UK. Between November to February, about 87% (1537/1760) of the M:A2S – carrying virus isolates belong to the B.1.1.7 lineage. During the same period of October 2021 to February 2021, M:M84T increased from 0.1% to 0.23% in the USA. Further location and date details of these variants can be found in Table S1. To assess the statistical significance of the upward trends of the frequencies of M mutations of interest in all publicly reported genomes over time, we performed the one-sided Cochran-Armitage test using month as the ordinal variable. The *p*-values ranged from 0.9999884 or non-significant to 1.33E-40 or very significant for the six mutations of interest (A2S: 0.9999884; F28L: 1.31E-09; I48V: 1.33E-40; I82T: 2.08E-39; M84T: 1.28E-08; V70L: 1.02E-09).

**Figure 5.**
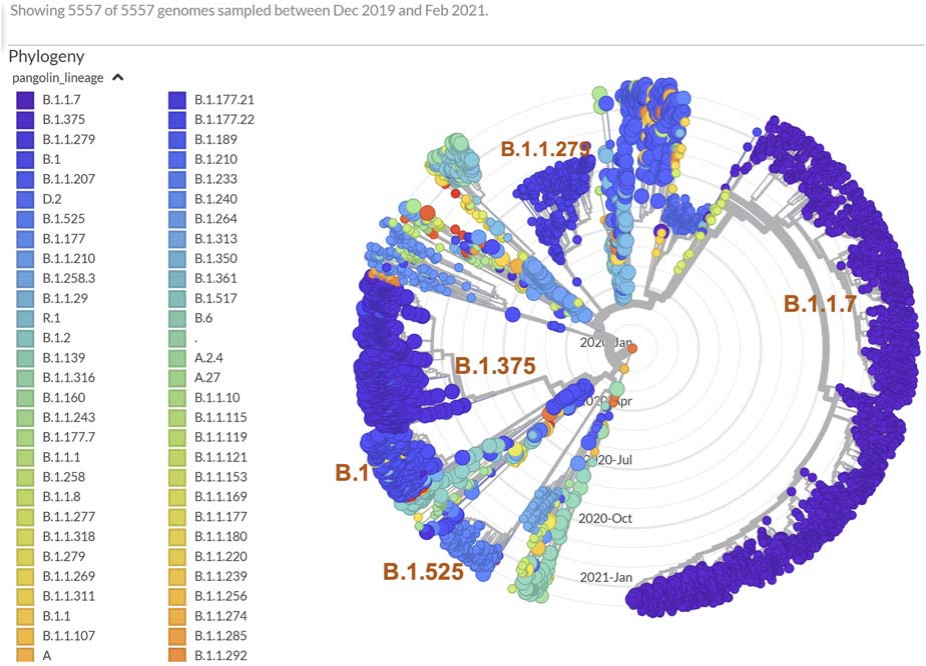
Phylogenetic analysis of viral genomes carrying missense M mutations, coloured by lineage background.

Focusing on the above-mentioned M mutations, we collected viral genomes from our own cases, GISAID, and GenBank that carried any of these mutations, as well as viral genomes that carried other M mutations by a haplotype similarity search which allowed a difference of up to five mutations across the genome thus were the likely ancestral or descendant isolates in evolutionary context. This yielded 5557 sequences that were analyzed for their phylogenetic relationships. The USA and UK sourced isolates were dominant in most clades, whereas limited mixtures exist in some cases (Supplemental Figure 1). Cross checking the country of origin and Pangolin lineage assignment, we observed that many of the isolates belong to the B.1.1.7 lineage, while most I48V mutation isolates belonged to lineage B.1.375. The timing of recent increases in both the number and ratio of the M-gene mutation carrying isolates in Europe, especially UK, were most likely closely related to the B.1.1.7 (Figure 5). The B.1.1.7 lineage also carries a synonymous M mutation, and hence significantly reduced the ratio of missense to synonymous mutations in worldwide isolates. The M:V70L and M:A2S mutations both stayed within a narrow range of 1.1% to 1.5% within the B.1.1.7 lineage between December 2020 and February 2021, suggesting that a hijacking effect may account for these observed changes. However, the surge in other M mutations, and the emergence of a potentially new sub-B1 M:I82T carrying clade exceed what would be expected by a hijacking effect alone.

**Figure 6.**
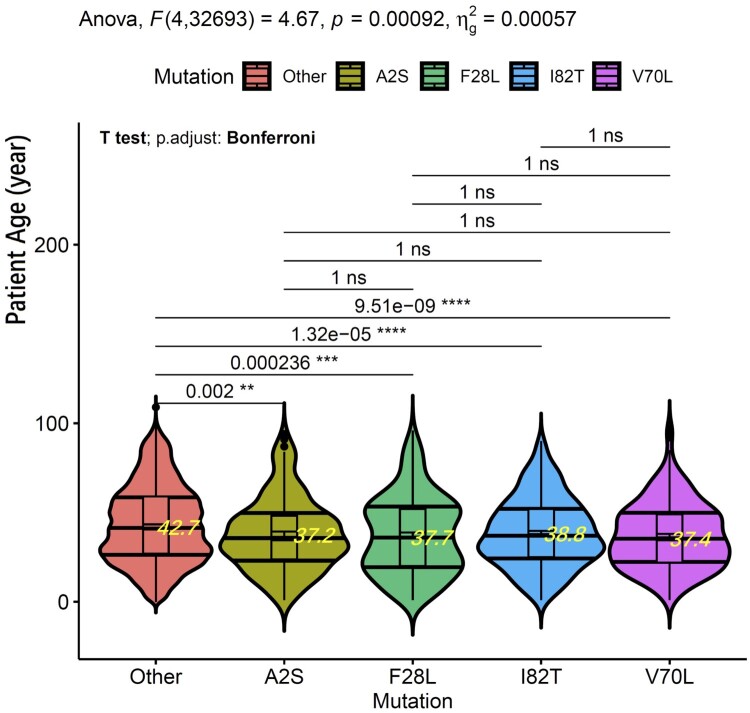
The ANOVA and *t*-test statistics between the five mutation groups. The plot was generated with R ggpubr package. Within each violin are the boxplot with error bars, and the horizontal lines at the quantiles 0.25, 0.50 and 0.75 of the density estimates. The pairwise *t*-test adjusted *p*-values and significance are shown to the upper part.

Patient age information was collected for cases reported between October and December 2020, and then in 2021, and compared between groups of cases carrying each of four M gene mutations versus the “other” group that does not carry any of the four mutations. The four mutations are M:A2S, M:F28L, M:I82T, and M:V70L. Each of the four M mutation groups has between 188 and 424 cases, and the “other” group has a total of 86,252 cases. One-way ANOVA analysis revealed significant difference in the patient age distribution among groups (*p* = 0.00092). Pairwise *T*-test, with Bonferroni multiple testing correction, indicates statistically significant patient age difference between each of the four M mutation carrying groups with the “other” group (adjusted *p*-values between 2.0e-4 and 9.5e-10), but not between each other (Figure 6). To avoid potential uneven sampling effect, we also downsampled the “other” group to 2000 randomly selected sequences. The pairwise *T*-tests again demonstrated significant patient age difference between each of the four M mutation carrying groups with the “other” group (adjusted p-values: “Other” vs. “A2S – 0.006; “Other” vs. “F28L” – 0.001; “Other” vs. “I82T” – 0.00035; “Other” vs. “V70L” – 6.24E-08). Patient ages of these four M-gene mutation groups were between 37.1 and 38.8 years on average, which were 4.6 to 6.3 years younger than the mean patient age of 43.4 years in the “other” group. We speculate that these M gene mutations may be associated with increased transmissibility among the younger population.

## Discussion

The M gene encodes the most abundant of three SARS-CoV-2 structural proteins, in this case a 222 amino acid protein that is highly conserved between SARS-CoV and SARS-CoV-2 (identity: 90.5%; similarity: 98.2%) [13]. Comparatively little attention has been paid to the M protein in the COVID-19 pandemic literature but it is known to be important for viral assembly, and in addition it markedly inhibits type I and III interferon production and thus dramatically inhibits the innate immune response [14, 15]. That in turn blunts the T-cell mediated immune response which is known to be important in overall immunity to SARS. In SARS due to SARS-CoV, the M protein is the dominant immunogen for T-cell response [16]. In COVID-19 due to SARS-CoV-2, T-cell response has been identified as a critical determinant of outcome, with poor T-cell response to M protein epitopes found in patients with fatal outcome [17, 18]. T-cell responses are a critical part of the successful immune response against emerging VOCs that may enable immune evasion [19, 20]. Therapeutic strategies that target the M protein and thus modulate T-cell responses have recently been proposed as promising alternatives to current ones [21].

Unfortunately, the protein structure of the M protein has not been experimental determined so that it is not possible to precisely predict the structural and functional impacts of these M mutations. The predicted SWISS-Model (PODTC5) of the M protein does not cover all the amino acids either. *In silico* analysis, however, revealed that the M protein structure was similar to that of the glucose transporter SemiSWEET with three transmembrane helical domains, based upon which the M protein is thought to be involved in enhanced glucose transport in host cells with replicating virus, and thus may aid in rapid viral proliferation, replication, and immune evasion [22]. The M:I82T mutation falls in the third transmembrane helical domain [22]. These transmembrane domains vary in number in the SWEET – 7, SemiSWEET – 3, and GLUT1 – 14 glucose transport family and are thought to bind and transport glucose, yet another function of the M protein that also initiates viral binding to the cell membrane heparin sulphate proteoglycan (via the N terminal exposed fragment), viral protein assembly via the internal carboxyterminal fragment, and immune evasion by inhibition of nuclear transport of NFκB signal transducers involved in interferon induction. Structural prediction, however, did not suggest significant impact of structural changes to be caused by the M:I82T mutation which is a hydrophobic to slightly polar amino acid change (Figure 2). According to Missense3D, this mutation does not change the secondary structure, nor does it introduce buried charge or hydrophilic. No buried H-bond breakage is induced, but a new H-bond is formed with GLY78 residue.

We have identified that the M gene, though otherwise highly conserved throughout most of the pandemic, is now undergoing rapidly increasing mutation with a recent surge in isolates carrying previously unreported M gene mutations in the USA and globally. In particular, we identified the emergence of a novel M:I82T clade over the last three months in the eastern USA. In addition, we note that the V70L undergoing rapid expansion in the UK and the I82T mutations, both of which are under increasing rapidly, involve the putative glucose transport transmembrane helices of the M protein. We continued to follow the frequency of isolates carrying the M:182T mutation during the development of the manuscript. While it has increased in the USA from 0.014% in October 2020 to 1.62% in February 2021, a 116-fold change, it has further increased to 3.33% in isolates reported between February 16th till March 4th 2021. Given the rapid emergence of this mutation, and the role of the M protein in multiple viral functions, including viral host cell binding, innate immune and T cell responses in SARS and SARS-CoV-2, with possible immune evasion, and now potential alterations in glucose transport, this novel M:I82T clade warrants inclusion in ongoing SARS-CoV-2 genomic surveillance and further evaluation for potential increased geographic spread and pathogenicity. Of particular interest is the observation that the average age of patients infected by the virus carrying one of the four M missense mutations is 4.6-6.3 years less than those that lack any of the M mutations (37.1–38.8 vs. 43.4 years of age). Given the increasing incidence of symptomatic and even severe COVID in younger patients, the potential for association with this mutation bears scrutiny.

## Supplementary Material

Figures_and_supplemental_figures.zip
