# Supplementary figures and images for: Emerging variants of concern in SARS-CoV-2 membrane protein: a highly conserved target with potential pathological and therapeutic implications
Source: Emerg Microbes Infect. 2021 May 9;10(1):885–93. doi: 10.1080/22221751.2021.1922097 (PMC8118436; doi:10.1080/22221751.2021.1922097)

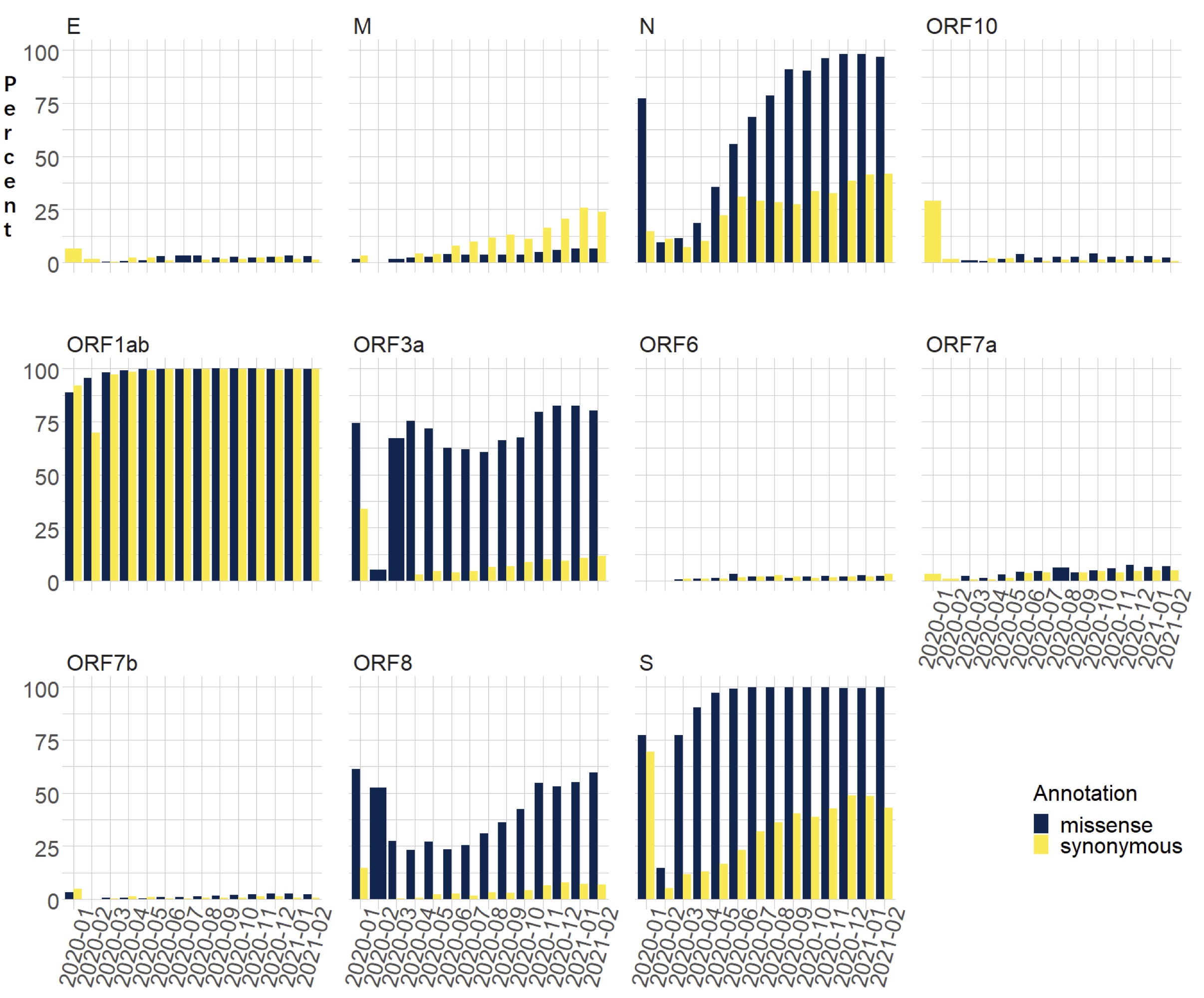

Supplement: Figures_and_supplemental_figures.zip [file TEMI_A_1922097_SM1844.zip › Figure1.jpg]

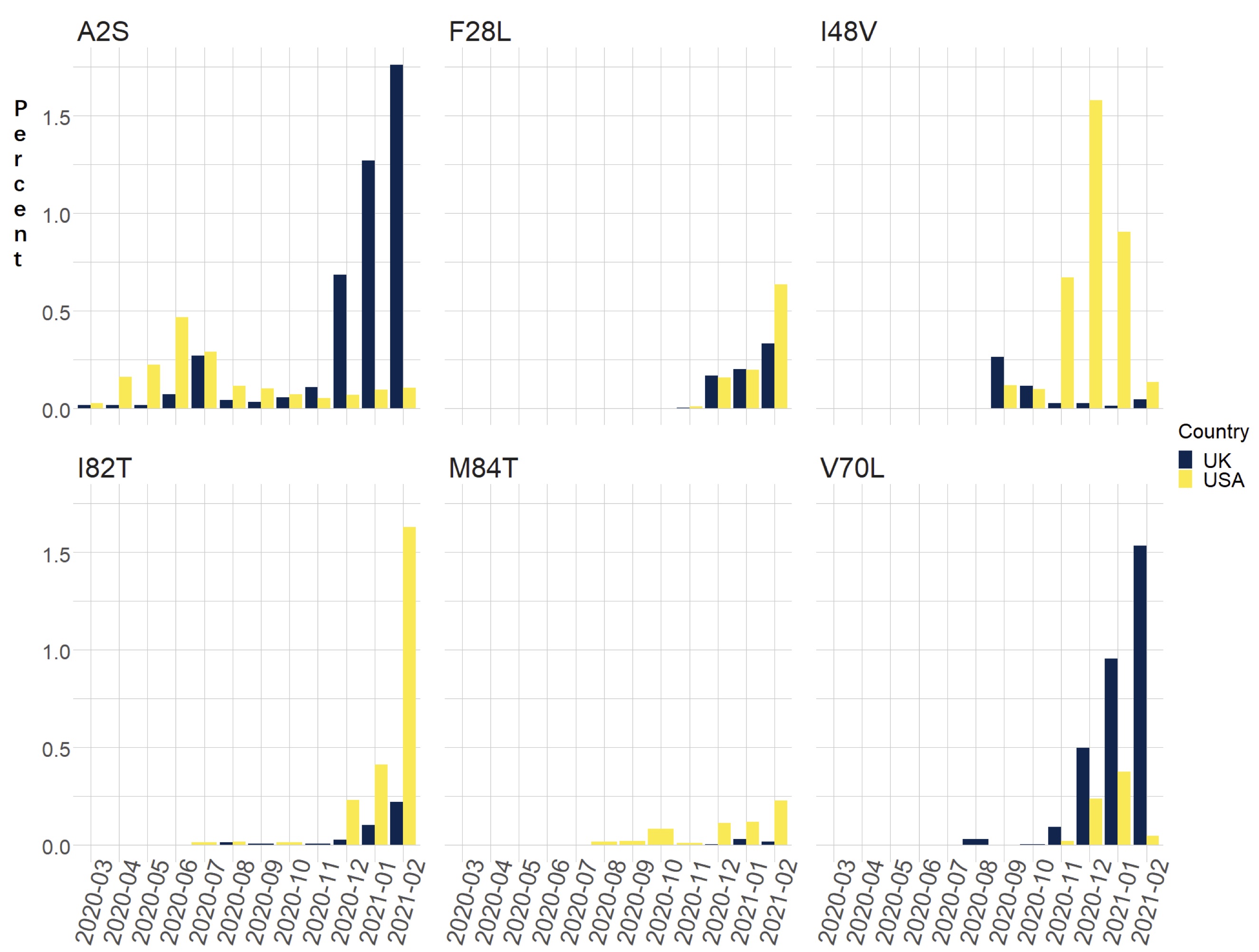

Supplement: Figures_and_supplemental_figures.zip [file TEMI_A_1922097_SM1844.zip › Figure2.jpg]

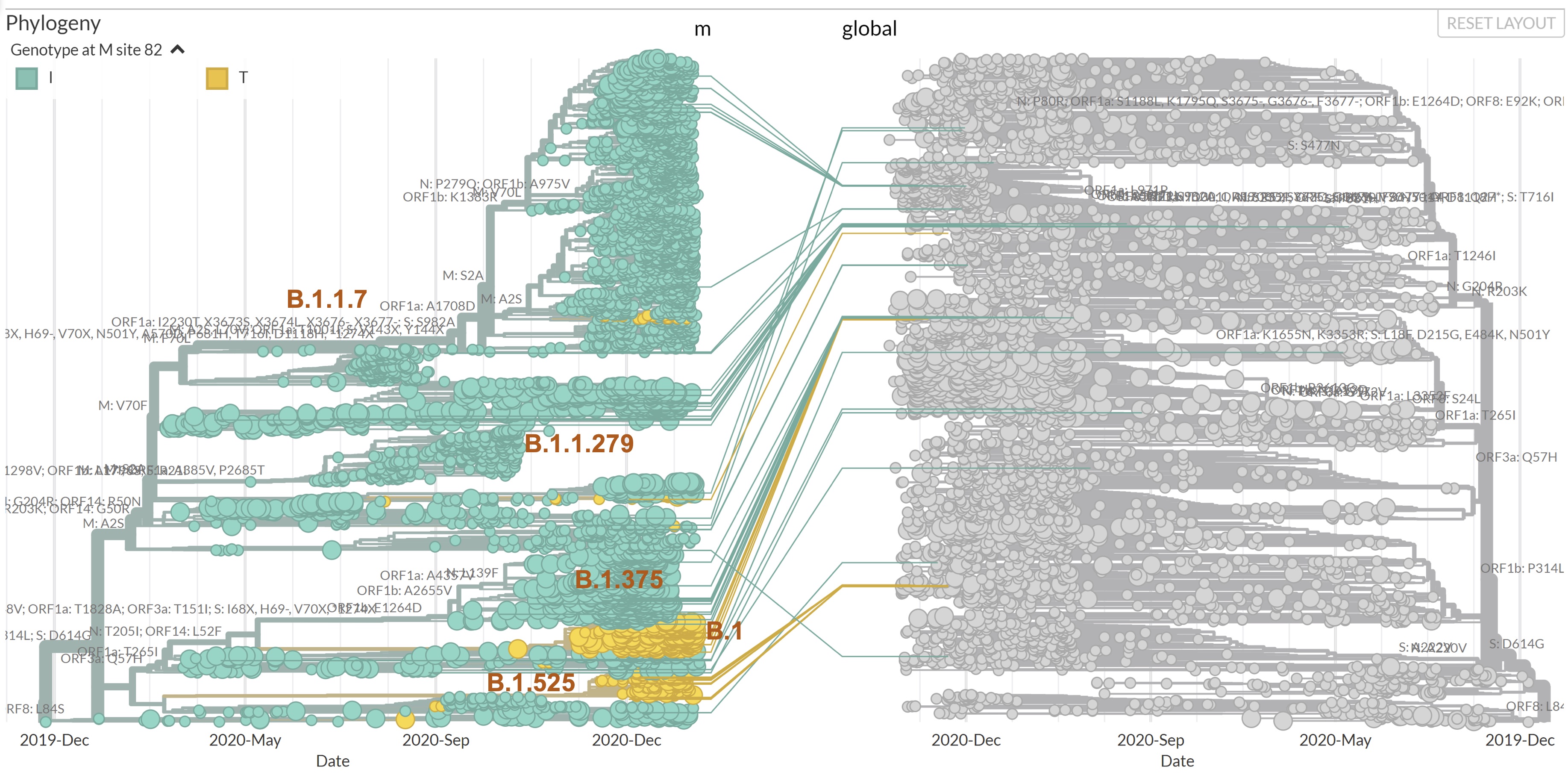

Supplement: Figures_and_supplemental_figures.zip [file TEMI_A_1922097_SM1844.zip › Figure3.jpg]

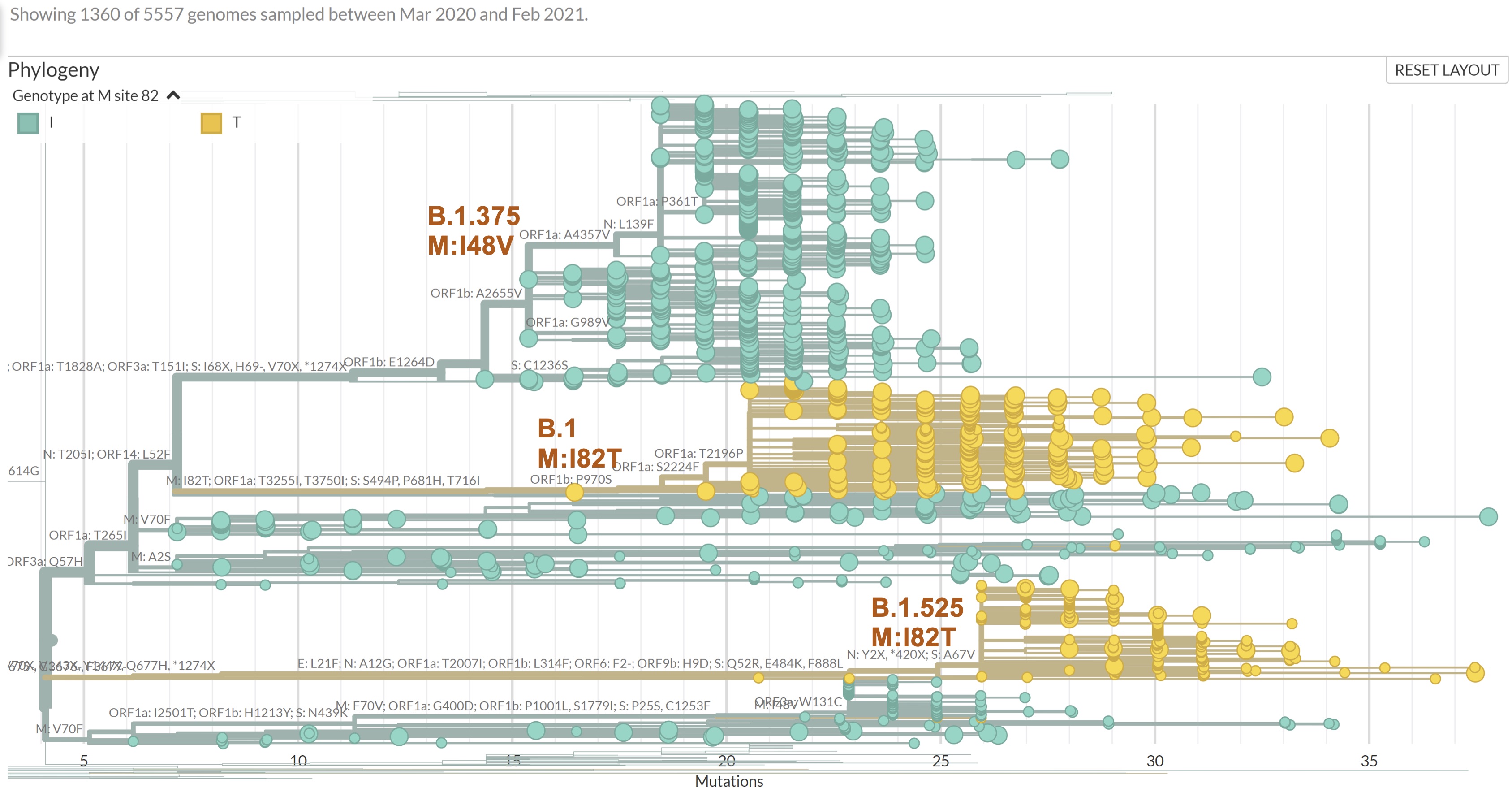

Supplement: Figures_and_supplemental_figures.zip [file TEMI_A_1922097_SM1844.zip › Figure4.jpg]

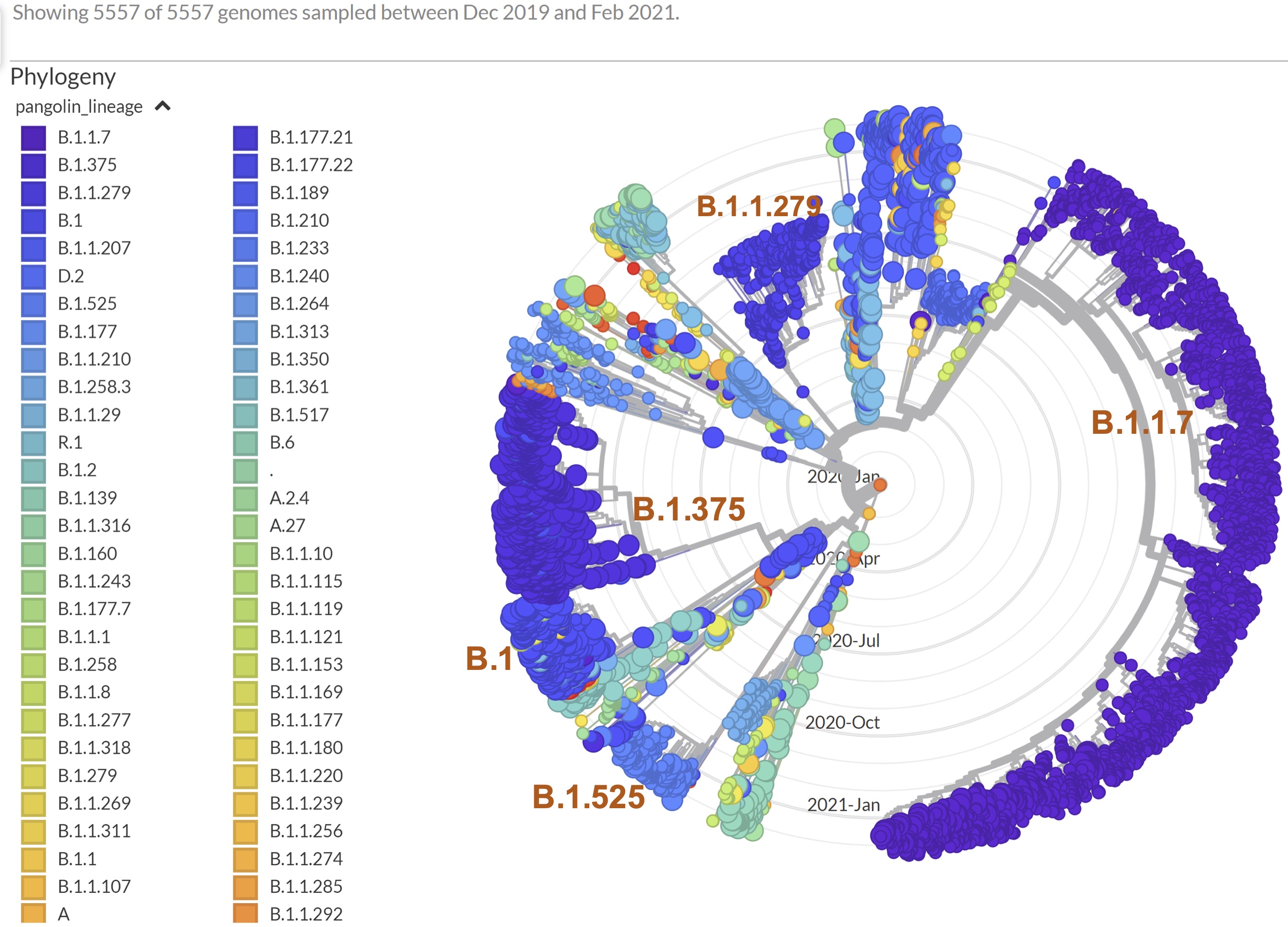

Supplement: Figures_and_supplemental_figures.zip [file TEMI_A_1922097_SM1844.zip › Figure5.jpg]

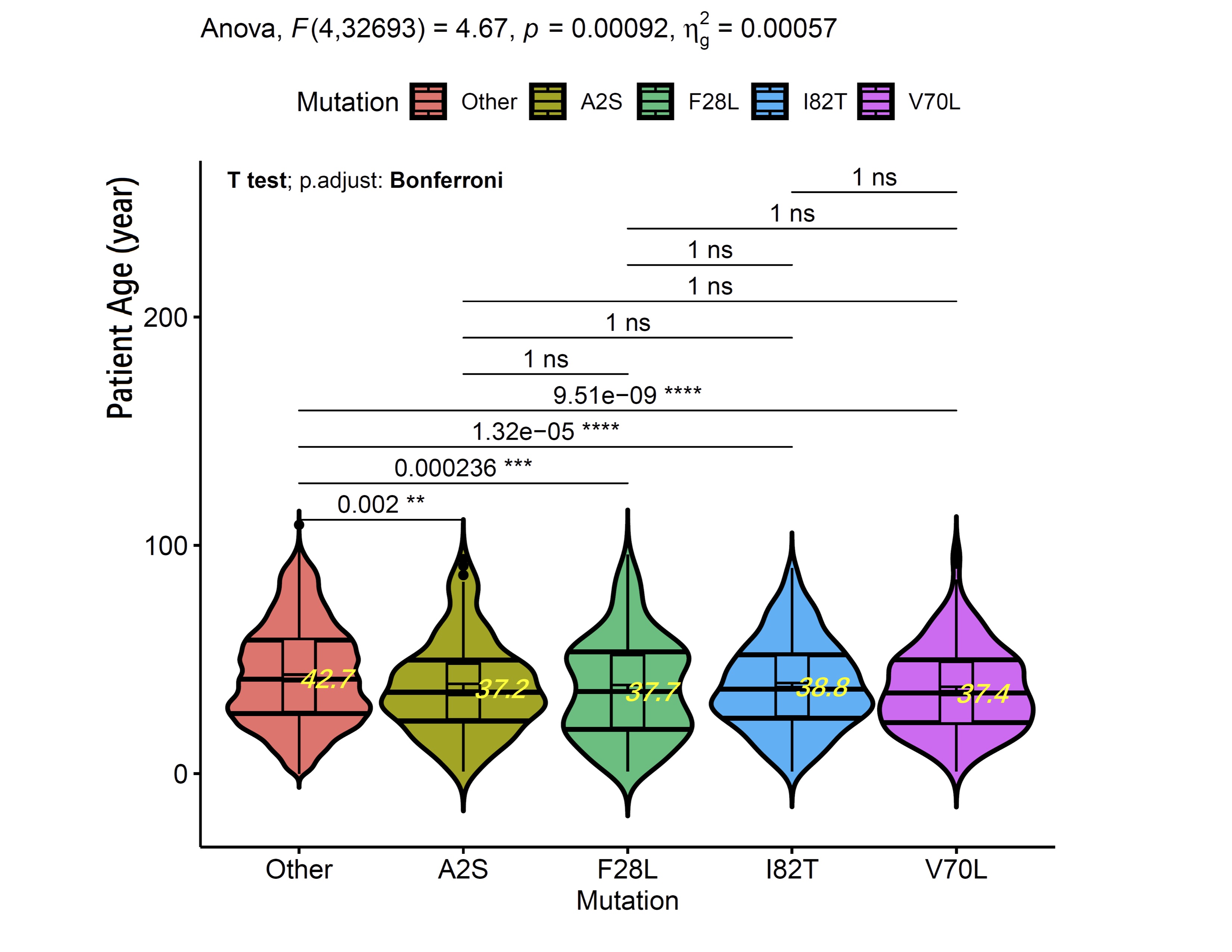

Supplement: Figures_and_supplemental_figures.zip [file TEMI_A_1922097_SM1844.zip › Figure6.jpg]

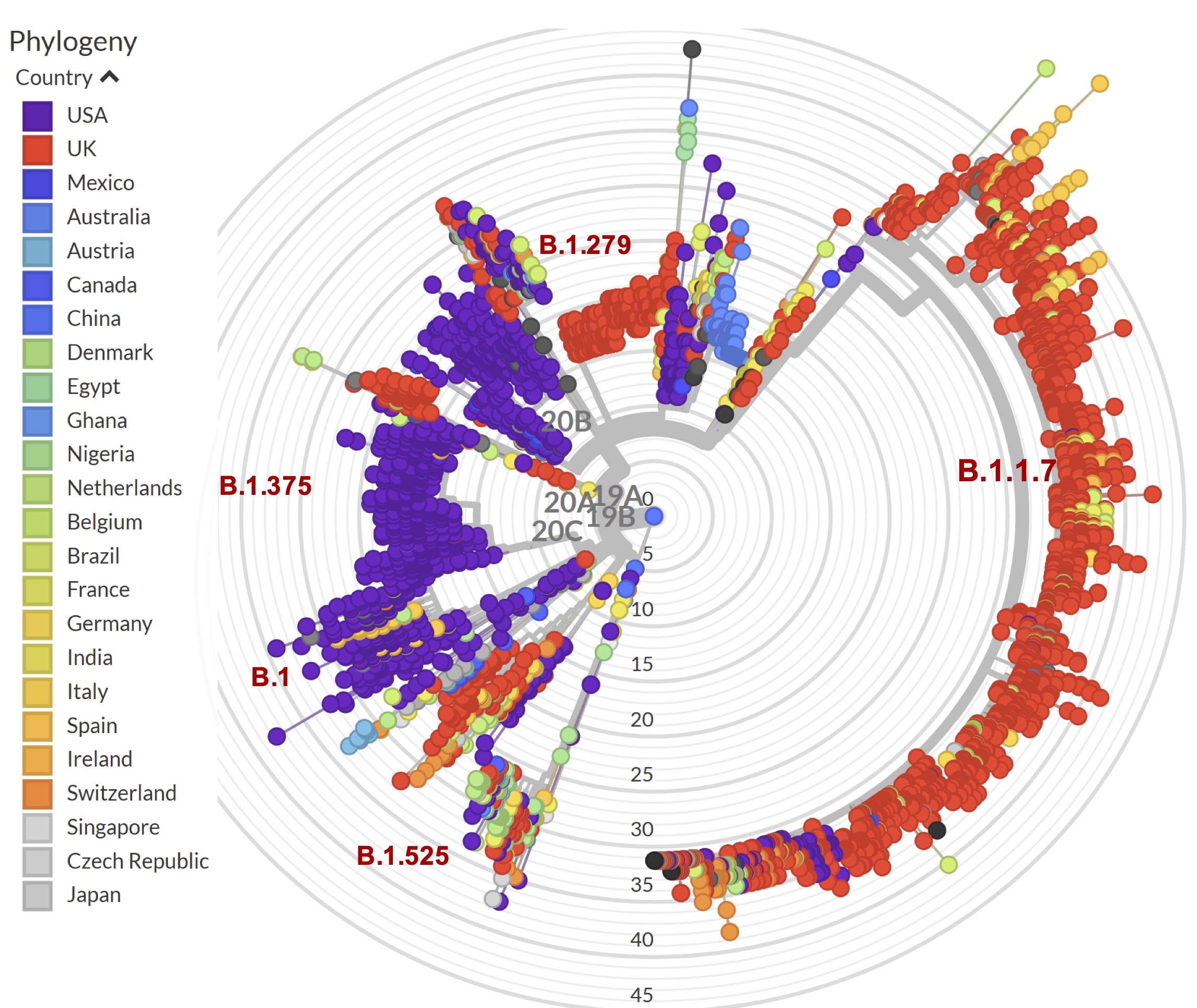

Supplement: Figures_and_supplemental_figures.zip [file TEMI_A_1922097_SM1844.zip › Supplemental_Figure1.jpg]

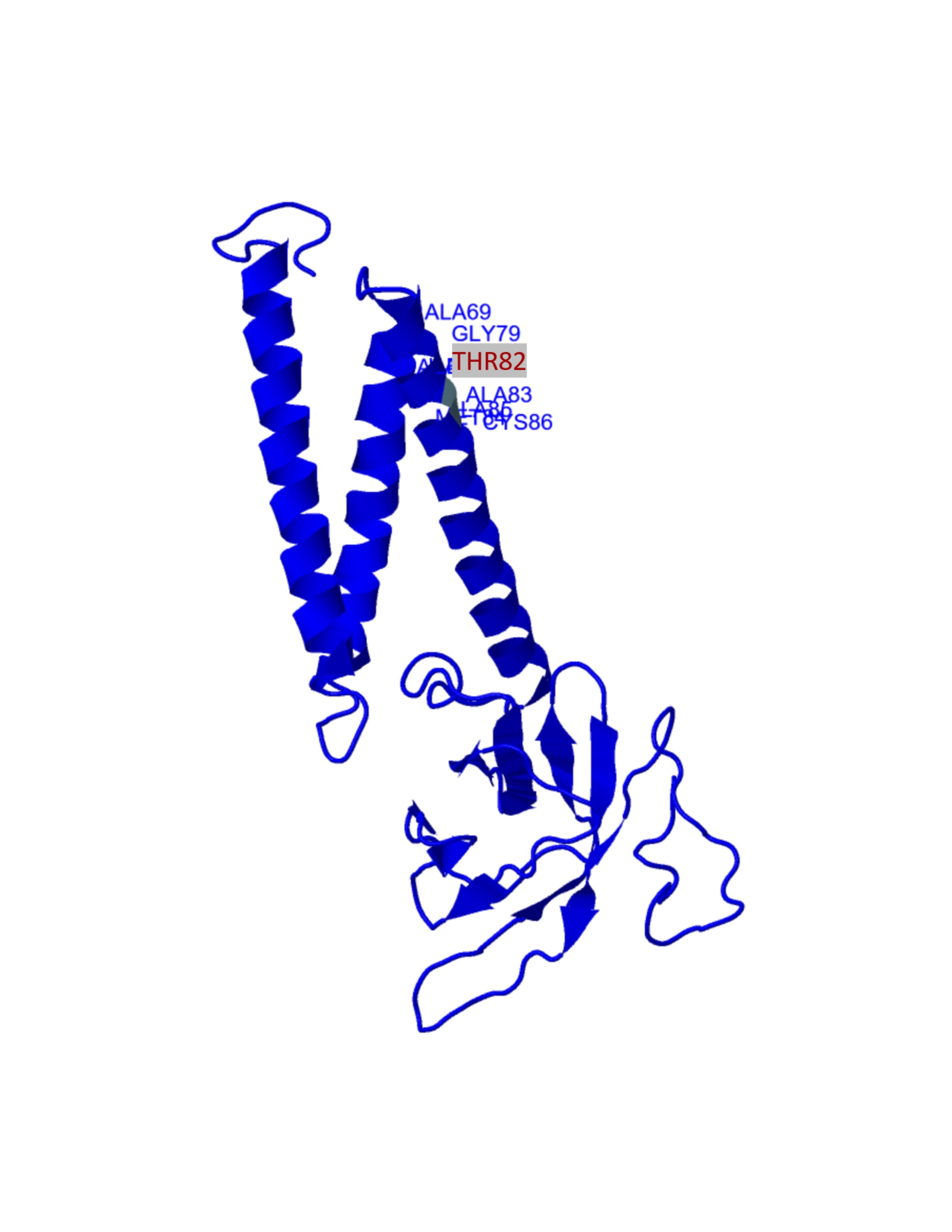

Supplement: Figures_and_supplemental_figures.zip [file TEMI_A_1922097_SM1844.zip › Supplemental_Figure2.jpg]
